# Unique Features of HIV-1 Spread through T Cell Virological Synapses

**DOI:** 10.1371/journal.ppat.1004513

**Published:** 2014-12-18

**Authors:** Raymond A. Alvarez, Maria Ines Barría, Benjamin K. Chen

**Affiliations:** Division of Infectious Disease, Department of Medicine, Immunology Institute, Icahn School of Medicine at Mount Sinai, New York, New York, United States of America; University of Florida, United States of America

The spread of viral infections can be initiated by the release of cell-free virus particles that infect at a distance or via cell-associated virus, which can promote the direct transmission of viruses between adjacent cells. In the case of human immunodeficiency virus type 1 (HIV-1), cell–cell contact has been found to enhance infection through specialized structures called virological synapses (VS). Cell–cell interactions between virus scavenging dendritic cells and T cells or between infected and uninfected T cells are two major cell interactions that enhance HIV infection. Here we review the features of VS formed between infected and uninfected T cells and focus on how these differ from infection by cell-free virus. While virus particle production is a shared characteristic of both cell-free and cell–cell HIV transmission, cell–cell infection displays several unique features that contribute to the enhanced efficiency of this mode of transmission. Five distinguishing features of HIV spread through T cell virological synapses are discussed below.

## Cell–Cell Adhesion Activates Virus Assembly at the VS

**Figure 1:**
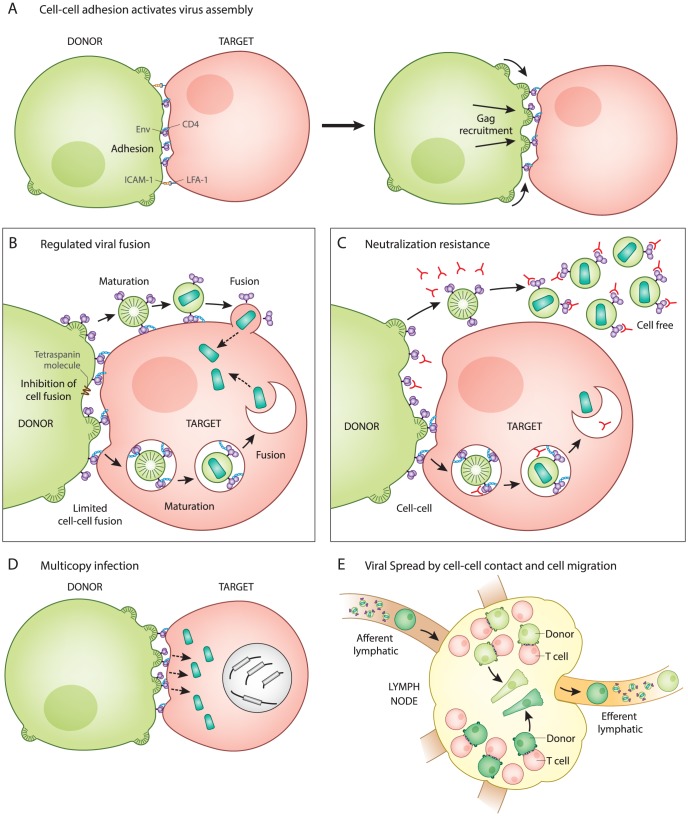
Five unique features of HIV-1 transmission through virological synapses. **A.** Cell–cell adhesion activates virus assembly at the VS. Infected T cells bind to uninfected T cells through an interaction between Env and CD4 (left). This is observed to occur prior to the recruitment of Gag to the site of adhesion (right). **B.** Regulation of Env-mediated membrane fusion. Cell–cell adhesion involves engagement of Env with CD4 at the surface of the infected and uninfected cell, but does not trigger cell–cell fusion. Host factors and the immature Gag lattice are two mechanisms that may prevent fusion until virus particles have budded from the donor cell and are transferred to the target cell, which has been observed as occurring through an endocytic route. Viral entry after synapse is likely to be activated by viral maturation rather than CD4 binding. **C.** Neutralization resistance. Cell-free virus is easier to neutralize (has lower IC50) than the same virus when it infects directly from cell to cell. The resistance may be due to regulation of Env conformation that inhibits membrane fusion on the cell surface and promotes Env-dependent viral membrane fusion only after virus particle maturation. **D.** Multicopy infection and drug resistance. Many viral particles are simultaneously transferred and can infect to high copy number in vitro. This may contribute to a shift in IC50 of some classes of antiretrovirals and can provide an explanation for how selective pressure on HIV-1 acts on cells that have co-inherited multiple sequences rather than individual sequences. **E.** Viral spread by cell migration and cell–cell interaction. Spatial clustering of genetic HIV-1 variants in human splenic tissue is represented by cells colored in different shades of green. Live imaging studies indicate that infected cells, while slower than their uninfected counterparts, are motile, as represented by elongated cells. Inhibition of T cell migration in humanized mice suggests that infection can be restricted to small tissue subcompartments, and that viral spread in some animal models requires the migration of T cells out of the draining lymph node.

The T cell VS was initially characterized as an actin-dependent polarization of viral proteins Gag and Env to the site of cell–cell contact on infected donor cells and the recruitment of viral receptor CD4 to the site on uninfected target cells [1]. During cell–cell transmission of HIV-1, an infected T cell first forms a stable adhesive junction with an uninfected CD4+ T cell, which serves as a focal point for de novo viral assembly and transfer [2]. VS formation can be described as a two-part process that begins with adhesion triggered by Env-CD4 interactions and is stabilized by interactions between cellular adhesion molecules (i.e., LFA-1 and ICAM-1,3) [3]. When presented on an artificial membrane in the presence of ICAM-1, HIV Env is sufficient to trigger the arrest of CD4+ T cell migration and initiate VS formation (Fig. 1A) [4].

After cell–cell adhesion, the signaling of the VS partly resembles immune signaling through immunological synapses (IS). The tyrosine kinase Zap70 that is involved in IS signaling also promotes the recruitment of Gag to the site of contact between cells in the VS, but without the involvement of the T cell receptor [5]. In addition, the budding of HIV at VS and release of secretory microvesicles at IS both appear to exploit similar machinery for the directional secretion and budding of particles at their respective synapses [6], [7]. When VS were imaged by live confocal microscopy, fluorescently tagged Gag proteins rapidly mobilize and concentrate at the site of cell–cell contact [2]. Time-lapse imaging showed that cell adhesion occurred before Gag was recruited to sites of cell–cell contact, indicating that Env functions first as a cell adhesion molecule even before it associates with the newly forming virus particles [2]. The mechanism by which Env engagement triggers the recruitment of Gag to the VS remains an important question.

## Regulation of Env-Mediated Membrane Fusion

While virus particle production is required for both cell-free and cell–cell infection [8], these two modes of transmission differ with regard to how and when Env-mediated membrane fusion is activated. Entry of cell-free HIV-1 occurs when the viral Env protein interacts with CD4 on the surface of uninfected cells, which directly triggers viral membrane fusion. In contrast, during cell–cell HIV-1 infection, the initial interaction between Env and CD4 occurs at the cell–cell junction and does not immediately activate Env-mediated fusion (Fig. 1B). The direct fusion of synapsed cells is thought be inhibited by the interactions of Env with the immature lattice of Gag at the VS [9]. Additionally, membrane proteins such as tetraspanins and actin-membrane organizing proteins such as ezrin, which are both concentrated at the VS, also inhibit cell–cell fusion [10], [11]. The absence of significant levels of syncytia during VS formation [12] implies that Env-mediated fusion is regulated during this process.

Given that fusion is regulated during synapse formation, it is important to consider how it is activated following the transfer of virus particles into target cells. In studies of cell-free virions, the uncleaved Gag lattice within immature particles can inhibit viral fusion by interacting with the Env cytoplasmic tail [13], [14]. During VS infection, viral assembly is tightly coordinated with transfer. Electron microscopy studies have captured nascent viral budding forms engaged with the target cell, both in vitro and in vivo in humanized mice [2], [15]. Live imaging studies examining viral transfer across the VS revealed a rapid, CD4-dependent, translocation of particles into a protected, trypsin-resistant, virus-containing compartment [2]. This process of internalization has been characterized by some as having several features of clathrin-dependent endocytosis [16], [17], while others have concluded the pathway is non-endocytic [1]. During VS transmission, we have proposed that the activation of fusion is not triggered by CD4, which is engaged early during VS, but, rather, by particle formation and subsequent maturation, which was observed as occurring within an intracellular compartment [18]. Through this mechanism, viral membrane fusion may be prevented from occurring prematurely, prior to the formation of mature virus particles. The pathway that the virus takes into the cell and how the fusogenicity of Env is regulated warrants further study and may play a role in understanding how cell–cell infection promotes immune evasion (discussed further below).

## Neutralization Resistance

Recent studies have generally found that HIV-1 transmission through VS is more resistant to neutralizing antibodies as compared to infections with cell-free virus [19], [20]. Because cell-surface HIV-1 Env must engage CD4 on the target cell to initiate the VS, it is likely that Env is available for binding by neutralizing antibodies (Fig. 1C). We suggest that it is unlikely that Env is hidden in a privileged synaptic space if it must bind to CD4 at the cell surface. An important feature of the neutralization resistance of the VS is that the magnitude of the resistance depends upon the epitope on Env that is targeted [19]–[21]. This may imply that the antibody epitopes exposed during cell–cell transmission are conformationally restricted as compared to virus particles. Interestingly, the deletion of the cytoplasmic domain of Env enhanced the neutralization of cell–cell infection, while having very modest impact on cell-free virus [20]. Because this domain of the viral glycoprotein modulates the fusogenicity of Env in response to virus particle maturation [13], [14], it may be that cell-surface Env assumes unique conformational states that allow it to resist neutralization. To fully understand the resistance of cell–cell transmission to neutralization, it will be important to define the differences in Env conformation on the cell surface versus the virion surface.

## Multicopy Infection and Selective Drug Resistance

During VS transmission, many virus particles can be observed to transfer simultaneously across single synapses (Fig. 1D) [2]. Experimentally, cell-to-cell infection results in a greater copy number of successful viral integration events than predicted by a Poisson distribution [22], [23]. Studies on HIV-infected patient splenocytes have found multiple proviral copies of HIV-1 (3–4) per infected human splenocyte when examined by in situ hybridization [24]. In vitro studies report an enhanced resistance of cell-to-cell transmission to certain classes of antiretroviral drugs, which is attributed to the high multiplicity of infection mediated by VS [25], [26]. This relative resistance was overcome with highly-active combination therapies [25]. Multicopy infection has also been observed in single cell PCR studies [27], leading us to consider how the presence of coinfected cells may impact viral fitness. When mutant viral sequences are co-inherited with wild-type sequences, then many defective genomes may contribute to the gene pool without themselves being replication-competent. This provides a physical basis for selection of HIV-1 on the level of quasispecies rather than individual clonal sequences [22]. Cell-to-cell transmission may therefore contribute to the fitness of viral quasispecies by enhancing the diversity of actively transmitted sequences.

## HIV-1 Spread by Cell Migration and Cell−Cell Interaction

In contrast to virus dissemination by cell-free particles, dissemination by cell-associated mechanisms is potentially dependent upon the migration, compartmentalization, and cellular interactions of infected cells. While it remains difficult to directly measure the relative roles of cell-free and cell-associated virus in viral dissemination in vivo, a number of studies indicate that cellular migration through tissues is important for HIV spread in vivo.

Within lymphoid organs such as the spleen, HIV-1 replication has been described as spatially segregated where viruses with distinct sequences segregated between neighboring follicles [24]. This spatial segregation of viral sequences may indicate that spread within a tissue compartment is predominantly local and dependent on cell proximity (Fig. 1E). A revealing study of acute HIV-1 transmission in humanized mouse models reported that T cell migration is critical to promote systemic spread of HIV-1 [28]. In this study, a local infection could be inhibited from spreading to distal sites by blocking lymphocyte egress from the draining lymph node using an inhibitor of the sphingosine monophosphate receptor (S1PR) [28]. Future studies are needed to determine the extent to which lymphocyte migration and VS contribute to the systemic spread of HIV-1.

## Overview

While the basic steps of the virus life cycle are conserved between cell-free and VS-mediated infection, the latter exhibits a unique temporal and spatial organization of virus production that is integrated with cell–cell signaling and coordinated with cell migration within the body. Given the intimate connections of the VS with the biology of T helper cells, a deeper understanding of the VS will be required to gain a full appreciation of how HIV-1 spreads and causes disease.
